# Solar-Driven Paired CO_2_ Reduction–Alcohol Oxidation Using Semiartificial Suspension, Photocatalyst Sheet, and Photoelectrochemical Devices

**DOI:** 10.1021/jacs.4c10519

**Published:** 2025-02-28

**Authors:** Motiar Rahaman, Carolina Pulignani, Melanie Miller, Subhajit Bhattacharjee, Ariffin Bin Mohamad Annuar, Rita R. Manuel, Inês A. C. Pereira, Erwin Reisner

**Affiliations:** † Yusuf Hamied Department of Chemistry, University of Cambridge, Lensfield Road, Cambridge CB2 1EW, U.K.; ‡ Instituto de Tecnologia Química e Biológica António Xavier (ITQB NOVA), Universidade NOVA de Lisboa, Oeiras 2780-157, Portugal

## Abstract

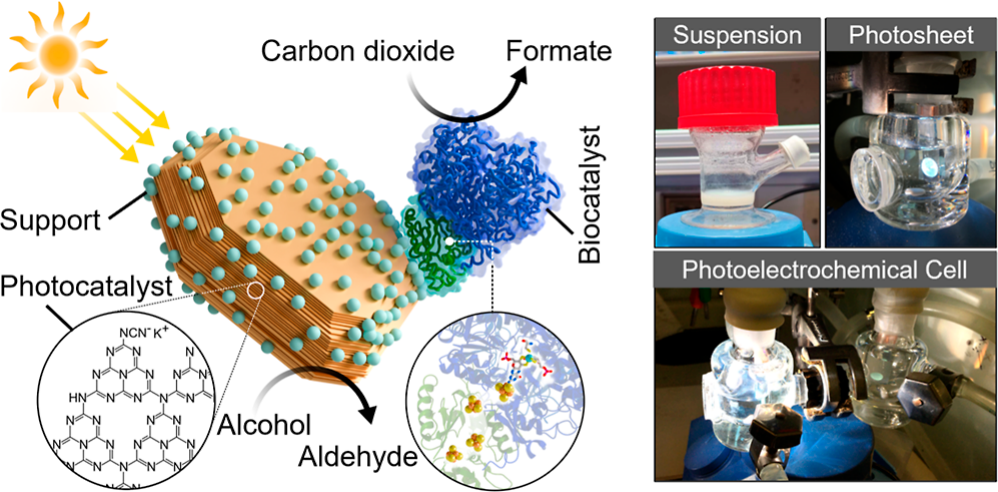

Fuel-forming enzymes can display excellent performance, achieving high rates of catalysis with unity selectivity at minimal overpotentials, but they are generally considered to be fragile and difficult to handle in combination with synthetic semiconductors in light-driven chemical synthesis. Here, we demonstrate a biohybrid platform that is assembled from cyanamide-functionalized carbon nitride (CN_*X*_) as a scalable and inexpensive photosensitizer that selectively photo-oxidizes 4-methyl benzyl alcohol (MBA) to its aldehyde (MBAld), indium tin oxide (ITO) nanoparticles as electron conduit and biocatalyst support material, and the enzyme formate dehydrogenase (FDH) for selective CO_2_-to-formate reduction. This integrated semiartificial multicomponent system can be assembled and used in several configurations to drive bias-free operation, including (i) a photocatalytic suspension, (ii) a photocatalyst sheet, and (iii) a photoelectrochemical cell. The unprecedented adaptability and robustness of the assembled biohybrid systems motivated us to select the best performing and scalable system in practical solar chemical production by deploying a 50 cm^2^ CN_*X*_–ITO|FDH photosheet device for 3 days under natural sunlight to produce 41 (mmol formate) m^–2^ and 35 (mmol aldehyde) m^–2^. We have therefore demonstrated that CN_*X*_–ITO|FDH provides a robust and versatile platform that enables solar chemical synthesis for several days in outdoor operation using natural sunlight.

## Introduction

Semiartificial assemblies, consisting of a synthetic light absorber and biocatalyst, have shown their promise for driving selective catalytic transformations efficiently using sunlight.^1^ The use of isolated enzymes in photocatalytic or photoelectrochemical systems benefits especially from their excellent selectivity and high rate of catalysis at minor overpotential.^2,3^ Metal-dependent formate dehydrogenase (FDH), in particular, W-FDH from *Desulfovibrio vulgaris* Hildenborough used in this study, has been established as a model bioelectrocatalyst as it reversibly interconverts CO_2_, protons, and electrons into formate when productively wired to an electrode or semiconductor surface.^4,5^ The surface reaction of immobilized FDH proceeds presumably through direct hydride transfer^6,7^ from the metal–thiol active site to the activated CO_2_ positioned in the second coordination sphere of the metal center.^8,9^ Thus, immobilized FDH enables mediator-free CO_2_/formate cycling at the thermodynamic potential, which enables optimal utilization of low-potential electrons generated by a photoexcited semiconductor.^10−12^

Formate is a useful CO_2_ reduction product as it can be used as a commodity chemical for different applications; for example, downstream (bio)chemical synthesis, as a liquid store to release H_2_ via dehydrogenation or as direct use in next-generation fuel cells.^13−15^ The economical synthesis of formate from the greenhouse gas CO_2_ using solar energy could thus contribute to establishing sustainable chemical processes. To source the electrons and protons required for CO_2_-to-formate reduction, the oxidation of alcohols (e.g., sourced from biomass and plastic waste) is a suitable alternative to water oxidation as it provides thermodynamic and economic advantages.^16,17^ 4-Methyl benzyl alcohol (MBA) is commonly used as a model substrate to demonstrate alcohol oxidation to 4-methyl benzaldehyde (MBAld).^18,19^

Carbon nitride is an inexpensive visible-light absorber that has been used in many photocatalytic reactions, including solar-fuel synthesis coupled to alcohol oxidations.^19,20^ This environmentally friendly and scalable photosensitizer is readily prepared from abundant precursors and has a band gap of about ∼2.7 eV with a band alignment that allows for fuel production, including H_2_ evolution and CO_2_ reduction in combination with a suitable cocatalyst.^21−23^ Recent advances demonstrated that photoelectrodes with cyanamide-functionalized carbon nitride (CN_*X*_) can be rationally designed with improved conductivity and charge-transfer efficiencies.^19,24,25^ Although metal nanoparticles,^26,27^ small-molecule catalysts,^28,29^ and enzymes such as hydrogenase^30,31^ have been used as a cocatalyst with CN_*X*_, their assembly with CO_2_-reducing enzymes such as FDH remains elusive due to difficulties in designing a functional CN_*X*_–enzyme interface.^32^ CN_*X*_ has been identified as a particularly active photocatalyst for selective MBA-to-MBAld oxidation,^19^ with the surface oxidation mechanism going through intramolecular proton transfer and cation-radical formation.^33,34^ Both MBA-to-MBAld and CO_2_-to-formate conversion are 2e^–^-transfer processes.

**Figure 1:**
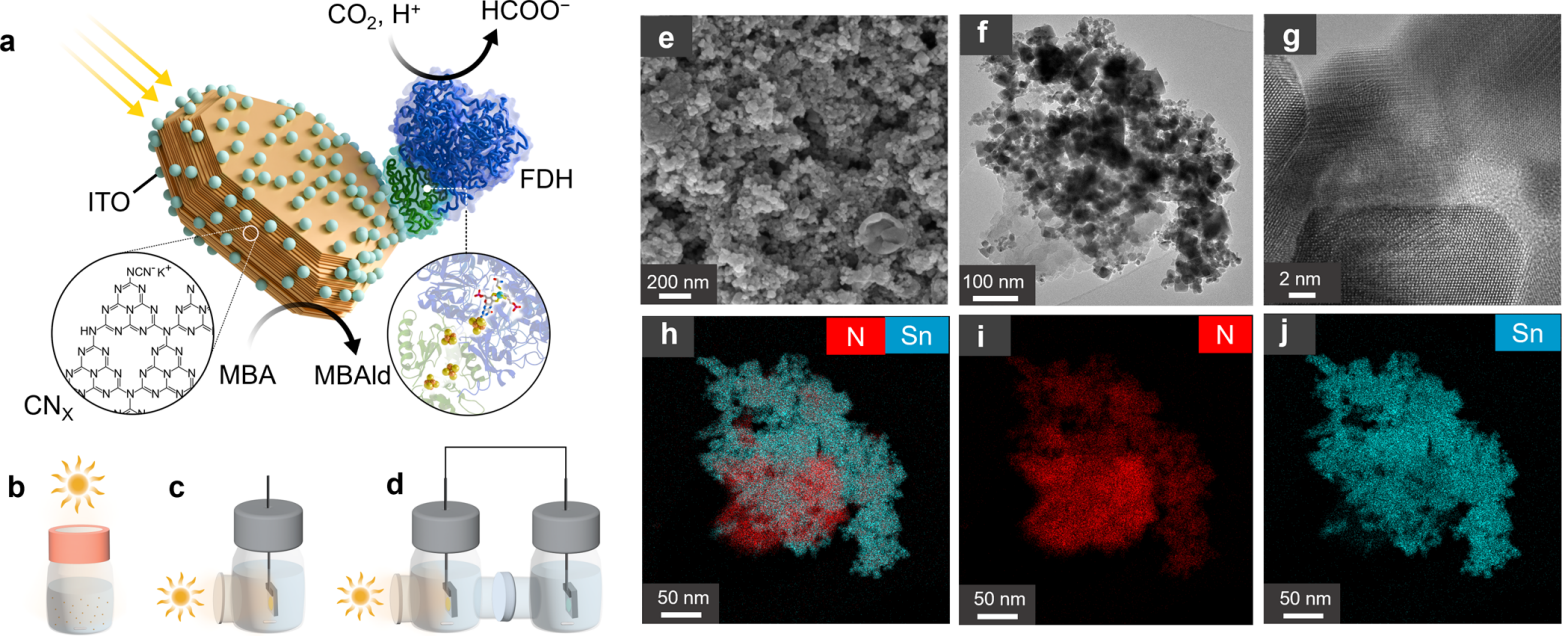
Photocatalytic process, device configurations, and material characterization. (a) Scheme showing the CN_*X*_–ITO|FDH biohybrid composite for solar-driven formate and MBAld production. Schematic of (b) top-down photocatalyst suspension setup, (c) single-compartment photocatalyst sheet setup, and (d) 2-electrode photoelectrochemical setup. (e) SEM, (f) TEM, and (g) HR-TEM analyses of the CN_*X*_–ITO (1:3) composite. (h–j) STEM–EDX mapping of the CN_*X*_–ITO (1:3) composite showing a homogeneous distribution of N and Sn over the material: combined N and Sn (h), N (i), and Sn (j).

Here, we report a semiartificial assembly consisting of FDH immobilized on a CN_*X*_–ITO (ITO = indium tin oxide) composite, where ITO acts as the support material to bind FDH and collects charge from the photoexcited CN_*X*_ (Figure 1a). The CN_*X*_–ITO|FDH biohybrid photoreduces CO_2_ to formate while oxidizing MBA to MBAld. The versatility of this integrated multicomponent system enabled the assembly of three different systems that were directly compared for solar-driven chemical production: (i) a photocatalytic suspension (Figure 1b), (ii) a photocatalyst sheet (Figure 1c), and (iii) an unassisted two-electrode, two-compartment photoelectrochemical cell (Figure 1d). The photocatalyst sheet demonstrated excellent performance and was thus selected for the assembly of a medium-scale prototype device that we deployed under natural sunlight for 3 days.

## Results and Discussion

### Synthesis and Characterization of CN_*X*_–ITO Composite

The synthesis of cyanamide-functionalized polymeric CN_*X*_ was carried out following a previously reported procedure.^19^ The ITO nanoparticles (<50 nm nanopowder, surface area 47 m^2^ g^–1^) were mixed with the CN_*X*_ at different weight ratios (3:1, 1:1, 1:3, and 1:9 for CN_*X*_/ITO, keeping the total mass constant) by sonication or grinding followed by a thermal annealing treatment under an Ar atmosphere. The different compositions were characterized using transmission electron microscopy (TEM, Figure S1) and UV–vis spectroscopy (Figure S2) analyses. Scanning electron microscopy (SEM) and TEM analyses of the CN_*X*_–ITO (1:3) photocatalyst composite show a mixture of pseudospherical, square, or octahedral particle morphology (Figures 1e,f and S3). High-resolution TEM (HR-TEM) analysis shows the ITO fringes (*d* = 0.505 nm; Figure 1g). Scanning transmission electron microscopy–energy dispersive X-ray diffraction spectroscopy (STEM–EDX) elemental mapping analysis reveals the distribution of N (from CN_*X*_) and Sn (from ITO) on the composite material (Figure 1h–j), which indicates an efficient connection between CN_*X*_ and ITO components, crucial for a compact composite that facilitates electron transfer processes. STEM–EDX analyses with different compositions show clear N and Sn distributions in accordance with their concentration (Figure S4). Powder X-ray diffraction (PXRD) shows the presence of graphite C and ITO peaks in the composite materials (Figure S5).

### General Considerations for Photocatalysis

The CN_*X*_–ITO|FDH biohybrid was employed in three different configurations (Figures 1b–d and S6), which are reported in detail below. In all systems, the photocatalytic reduction of CO_2_ to formate is coupled with the oxidation of MBA into its corresponding aldehyde. The band energies of the CN_*X*_ photocatalyst (valence band = 2.2 V; conduction band = −0.45 V vs the reversible hydrogen electrode, RHE at pH 7)^35^ align wih the thermodynamic reduction potential of CO_2_ reduction over FDH (−0.01 V vs RHE at pH 7, which also reflects the onset potential for FDH catalysis)^10,36,37^ and thus ensure the overall thermodynamic feasibility of the process in the biohybrid assembly under solar irradiation. The ITO nanoparticles function as a support for the biocatalyst as well as a conductor to transfer electrons from CN_*X*_ to FDH (Figure S7).

To the best of our knowledge, this study reports for the first time the use of the same photocatalytic system to assemble a photocatalyst suspension, a photocatalyst sheet, and a photoelectrochemical cell, which shows the possibility to interchangeably use materials developed for one configuration in another design. Ultimately, this has been made possible by the robust and versatile biohybrid assembly used here, which allows to readily couple and decouple the light absorber, electron relay, and catalytic unit.

Despite the robustness in operation, we note that the handling and attachment of FDH as well as the reactor assembly were exclusively performed inside a glovebox under an inert (N_2_) atmosphere to avoid damage of the enzyme. All light experiments were conducted under an O_2_-free atmosphere to prevent O_2_ reduction and damage of components from reactive oxygen species. A CO_2_-saturated NaHCO_3_ solution (100 mM) containing KCl (50 mM) and MBA (7.5 mM) at pH 6.7 was used as the reaction medium for the photocatalyst suspension, photocatalyst sheet, and scalable prototype photosheet experiments. For the photoelectrochemical experiments, the cathode compartment of the photoelectrochemical cell contained a CO_2_-saturated NaHCO_3_ solution (100 mM) with KCl (50 mM) at pH 6.7, where the same solution was used in the photoanode compartment after addition of MBA (7.5 mM). The photoexperiments were performed under 1 sun irradiation (AM1.5G, 100 mW cm^–2^) using a solar-light simulator, and the reaction temperature was maintained at 30 °C for all three systems. The prototype scalable photosheet was studied outdoors under anaerobic conditions, where natural sunlight intensity and the local temperature were constantly monitored. Formate was quantified by ion-exchange chromatography and MBAld by high-performance liquid chromatography analysis. The headspace of the reactors was also analyzed by gas chromatography (to identify potential gaseous byproducts such as H_2_).

### Photocatalyst Suspension

The photocatalyst suspension was prepared by grinding CN_*X*_ and ITO nanoparticles (using different ratios, see below), followed by annealing of the mixture at 250 °C for 1 h under Ar. FDH (40 pmol) was then immobilized on the CN_*X*_–ITO (4 mg) composite powder inside a top-down photoreactor (Figure 1b) and then 2 mL of a CO_2_-saturated reaction solution (see above for composition of the reaction medium) was added to the photoreactor to make a suspension. The headspace of the reactor was further purged with CO_2_ before the experiment. The suspension was irradiated by a simulated solar simulator (AM1.5G, irradiation area of ∼2.5 cm^2^) for 10 h. The CN_*X*_–ITO|FDH biohybrid composites were active toward photocatalytic CO_2_-to-formate reduction, whereas the exclusion control experiments (CN_*X*_|FDH without ITO, ITO|FDH without CN_*X*_, and CN_*X*_–ITO without FDH, Table S1) showed no photocatalytic activity. Thus, all three components of the biohybrid material are essential and serve the proposed function, with the CN_*X*_ acting as a light absorber and alcohol oxidation catalyst, ITO allowing for adsorption of FDH and acting as electron relay avoiding the electrostatic repulsion between FDH and CN_*X*_, and FDH performing CO_2_ reduction.

**Figure 2:**
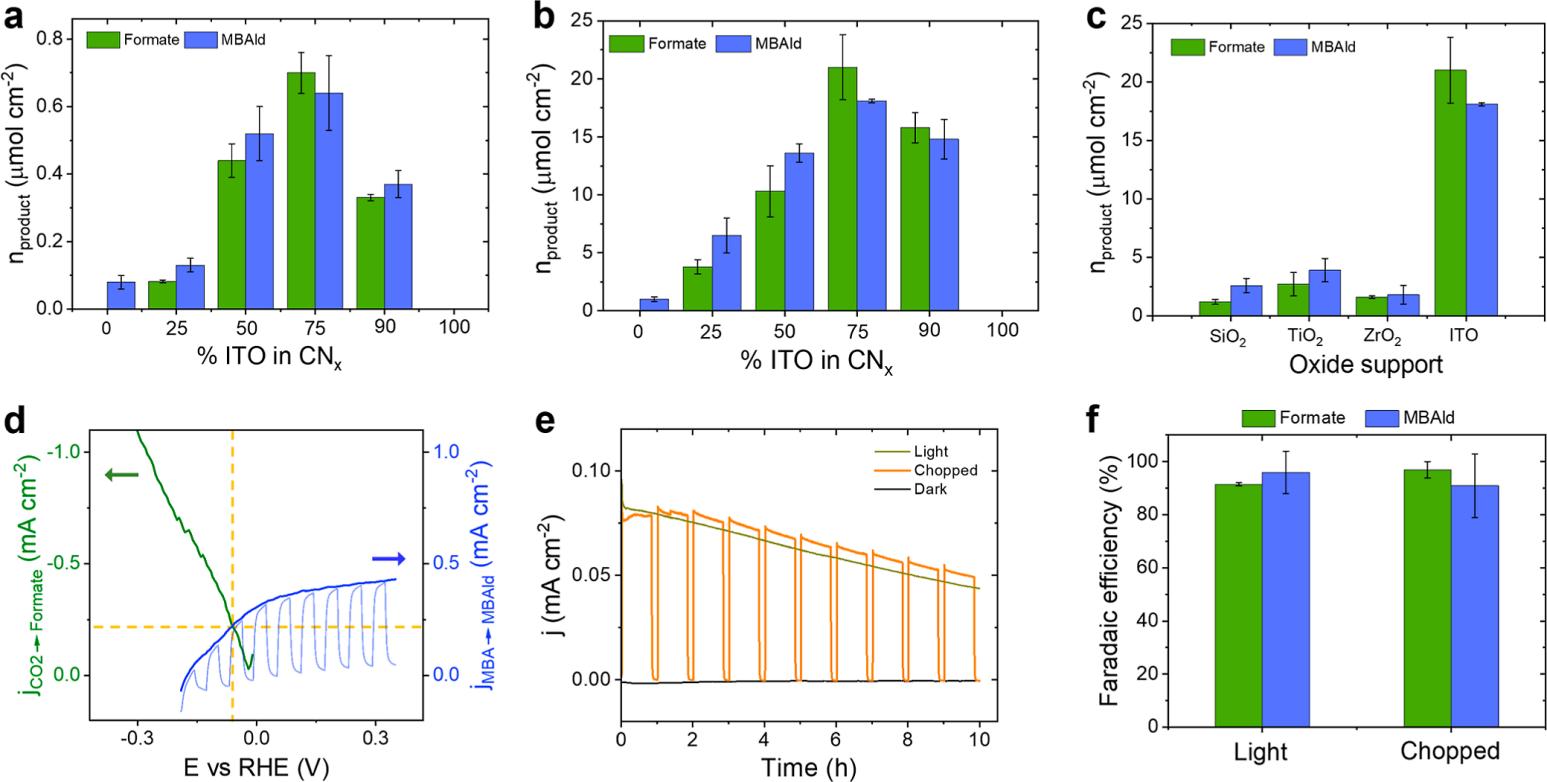
Performance comparison of CN_*X*_–ITO|FDH biohybrid systems. (a) Performance of the CN_*X*_–ITO|FDH photocatalytic suspension with different compositions, showing areal activity toward formate and MBAld production. (b) Composition-dependent activity of different CN_*X*_–ITO|FDH photocatalyst sheets. (c) Effect of different oxide supports on the activity of photosheet biohybrid systems. (d) Electrochemical–photoelectrochemical characterization of individual redox process: Overlap linear sweep voltammetry (LSV) scans of electrochemical CO_2_ reduction and photoelectrochemical MBA oxidation processes (scan rate ν = 10 mVs^–1^) using three-electrode configurations. The cathodic current (green trace) is shown inverted to illustrate the current crossover with the photoanodic response (blue trace). (e) Photocurrent density of a two-electrode CN_*X*_–ITO||IO–ITO|FDH system under chopped (50 min on, 10 min off), continuous, and no simulated solar-light irradiation under zero applied bias voltage. (f) Faradaic efficiency of formate and MBAld production from the photoelectrochemical system. Conditions: CO_2_-saturated NaHCO_3_ solution (100 mM) containing KCl (50 mM) and MBA (7.5 mM) at pH 6.7 was used as the reaction medium for both suspension and photosheet experiments. For the electrochemical LSV analysis under a CO_2_ reduction process, a CO_2_-saturated NaHCO_3_ solution (100 mM) with KCl (50 mM) at pH 6.7 was used as a catholyte, and for the photoelectrochemical LSV under the MBA oxidation process, the same reaction medium (pH 6.7) was used as an anolyte in the presence of 20 mM MBA. For the photoelectrochemical experiments, a CO_2_-saturated NaHCO_3_ solution (100 mM) with KCl (50 mM) at pH 6.7 was used as a catholyte and CO_2_-saturated NaHCO_3_ (100 mM), KCl (50 mM), and MBA (7.5 mM) at pH 6.7 was used as an anolyte. All reactions were performed under constant stirring and 1 sun (AM1.5G, 100 mW cm^–2^)-simulated solar irradiation at 30 °C.

The maximum photocatalytic activity was reached with a CN_*X*_/ITO ratio of 1:3 (Figure 2a), resulting in 0.70 ± 0.06 μmol_formate_ cm^–2^ (normalized to the irradiation area of the top-down photoreactor) and 0.64 ± 0.11 μmol_MBAld_ cm^–2^ after 10 h AM1.5G irradiation. This activity corresponds to a specific activity of 0.42 ± 0.04 mmol_formate_ (g of CN_*X*_–ITO)^–1^ and 0.38 ± 0.07 mmol_MBAld_ (g of CN_*X*_–ITO)^–1^ (Figure S8). A trace amount of MBAld production (<0.1 μmol cm^–2^) was observed on pure CN_*X*_ (in the absence of ITO) due to its charge-storing capability under light irradiation (Figure 2a).^19^ The stoichiometric ratio of formate and MBAld is ∼1:1, as expected from the two-electron nature of both half-reactions.

Significant photo-oxidation of accumulated formate (back reaction) over the CN_*X*_–ITO composite was ruled out by performing control experiments using a CN_*X*_–ITO|H_2_ase biohybrid (H_2_ase = NiFeSe-hydrogenase from *Desulfovibrio vulgaris* Hildenborough; see the Experimental Section for assembly details). The functionality of CN_*X*_–ITO|H_2_ase was confirmed in the presence of MBA as an electron donor, with substantial amount of H_2_ being produced (Figure S9). In the absence of MBA (water) or in the presence of formate, only trace amounts of H_2_ were produced after 8 h of AM1.5G irradiation. Thus, water and formate are not suitable as effective electron donors and demonstrate that MBA oxidation is dominating over a potential back reaction in the CN_*X*_–ITO|FDH biohybrid.

### Photocatalyst Sheet

The photocatalytic performance of the CN_*X*_–ITO|FDH powder system motivated us to prepare a photocatalyst sheet. Photocatalyst sheets are emerging as easily scalable systems for efficient solar fuel synthesis, particularly in the field of overall water splitting.^38,39^ We intended to develop a novel biohybrid photocatalyst sheet for combined CO_2_ reduction and alcohol oxidation processes. A mixture of CN_*X*_–ITO powder was dispersed in ethanol and dropcast (20 μL from a 45 mg mL^–1^ solution) onto a fluorine-doped tin oxide (FTO)-coated glass substrate (0.5 cm^2^ active area with ∼3 μm thickness of the CN_*X*_–ITO film) followed by annealing at 250 °C for 1 h under Ar.^24^ The gravimetric analysis confirmed that ∼400 μg of the CN_*X*_–ITO composite was deposited on the FTO substrate. FDH (200 pmol) was dropcasted onto the CN_*X*_–ITO photocatalyst sheet (0.5 cm^2^) before it was dipped into the 10 mL of CO_2_-saturated solution (see above for composition) in a single-compartment solar reactor (Figure 1c). The FDH-decorated photocatalyst sheets produced formate and MBAld in similar molar quantities in all experiments, consistent with the selective generation of both products.

The composition of the photocatalyst sheet was optimized by changing the CN_*X*_ and ITO ratio in the composite material, and a composition-dependent activity was observed (Figure 2b). The maximum photocatalytic activity was obtained again with a CN_*X*_/ITO ratio of 1:3, which produced 21 ± 3 μmol_formate_ cm^–2^ and 18 ± 0.1 μmol_MBAld_ cm^–2^ with a specific activity of 26 ± 3.5 mmol_formate_ (g CN_*X*_–ITO)^–1^ and 22.6 ± 0.2 mmol_MBAld_ (g CN_*X*_–ITO)^–1^ after 10 h AM1.5G irradiation. The FDH loading was then varied from 50 to 300 pmol on the optimized CN_*X*_/ITO (1:3) composite, resulting in a steady increase in activity up to 200 pmol whereupon a constant product formation rate was observed (Figure S10). Thus, 200 pmol FDH was identified as the optimal enzyme loading on the photocatalyst sheets. The optimized CN_*X*_–ITO|FDH biohybrid was also assembled on different glass substrates to study its catalytic activity. The biohybrid on the ITO-coated glass showed similar activity compared to the FTO-coated glass, whereas a lower performance was achieved on frosted glass, which is possibly due to mechanical instability of the CN_*X*_–ITO composite film (Figure S11). The FTO-coated glass was chosen as the primary substrate for biohybrid fabrication due to its relative cost and robustness.

The role of conducting ITO in the CN_*X*_–ITO composite was further investigated by replacing it with insulating and semiconducting metal-oxide particles. Photocatalyst sheets made of CN_*X*_–MO_2_|FDH with M = Zr, Si, or Ti using a CN_*X*_/MO_2_ ratio of 1:3 were assembled and assessed for their photocatalytic activity (Figure 2c). Very low activity was observed with the insulators ZrO_2_ and SiO_2_ (∼1.5 to 2 μmol_formate_ cm^–2^) after 10 h AM1.5G irradiation, whereas the wide-band gap semiconductor TiO_2_ also showed low activity (∼3 ± 0.7 μmol_formate_ cm^–2^) for CN_*X*_–TiO_2_|FDH. A 405 nm UV filter was used to prevent light absorption from the TiO_2_ semiconductor. Thus, using conducting ITO in the composite with CN_*X*_ enables the highest photocatalytic activity of the CN_*X*_–ITO|FDH system, supporting the importance of ITO to act as a solid-state electron conduit with CN_*X*_,^24^ in addition to its ability to strongly bind FDH.^40^

The reusability and stability of the CN_*X*_–ITO|FDH photocatalyst sheet were next studied by performing five cycles with the same photocatalyst sheet, where the CN_*X*_–ITO (1:3) was washed with water and loaded with fresh FDH after each 10 h AM1.5G irradiation cycle (Figure S12). The activity of the second cycle is comparable to the first cycle, but the activity started to markedly decrease in the third and fourth cycle (ca. 50% and 25% formate production activity in the third and fourth cycle, respectively, compared to first cycle). The photocatalyst sheet was then ozone cleaned after the fourth cycle to remove any potential residual deposits from protein degradation. However, the photocatalytic activity did not recover in the fifth cycle, indicating that residual deposits are not the main reason for losing activity. Postexperiment characterization of the photocatalyst sheets (after the first 10 h light irradiation) by SEM showed cracks on the surface and TEM/STEM-EDX analyses showed agglomeration of the photocatalyst nanostructures, indicating morphological changes of CN_*X*_–ITO to be a possible explanation for the loss in performance (Figures S13 and S14).

Exclusion control experiments by systematically removing one component of the photocatalyst sheet at a time showed that all components were required for photocatalytic activity (Table S1). The origin of formate was confirmed by isotopic labeling experiments, using ^13^CO_2_ and NaH^13^CO_3_ in the experiment. The ^13^C-labeled formate product (H^13^CO_2_^–^) was obtained with a clear doublet peak (*J*_C–H_ coupling = 188 Hz) at 8.34 ppm in the ^1^H-NMR spectrum, whereas only a singlet is observed at 8.34 ppm for H^12^CO_2_^–^ when using ^12^CO_2_ and NaH^12^CO_3_ in the standard experiments (Figure S15).

### Photoelectrochemical Cell

Following the demonstrations of CN_*X*_–ITO|FDH in suspension and photocatalyst sheet assemblies, we developed a photoelectrochemical platform with those components. A CN_*X*_–ITO photoanode (1:3 w/w %, deposited on an FTO-coated glass substrate, 0.5 cm^2^ active area, ∼3 μm film thickness) based on a previous preparation method was employed for MBA oxidation.^24^ An inverse-opal (IO) ITO cathode^41,42^ previously optimized for enzyme adsorption was prepared by depositing ITO nanoparticles with polystyrene beads (750 nm diameter) on an ITO-coated glass substrate (0.25 cm^2^ active area, see Methods for the detailed preparation procedure). FDH (100 pmol) was then dropcast on the IO–ITO scaffold to assemble an IO–ITO|FDH cathode for the CO_2_ conversion. A two-compartment cell with a proton exchange Nafion^117^ membrane separating the electrodes was used (Figure 1d).

The feasibility of the overall standalone operation of a two-electrode cell was first assessed by characterizing the individual photoanode and cathode using a three-electrode setup with a Pt mesh counter electrode and a Ag/AgCl (saturated NaCl) reference electrode. In agreement with previous reports,^10,43^ the IO–ITO|FDH cathode displays an onset of electrocatalytic formate production at a marginal overpotential (−0.1 V vs RHE) with almost 100% selectivity (Figure S16). The LSV with the CN_*X*_–ITO photoanode shows efficient catalytic activity for MBA oxidation providing an onset of −0.2 V vs RHE with a good fill factor and a steady-state current density of approximately 0.45 mA cm^–2^ (no photocurrent response under dark conditions; Figure S17). A combined plot with the LSV scans recorded under electrochemical CO_2_ conversion over the IO–ITO|FDH cathode and photoelectrochemical MBA oxidation over the CN_*X*_–ITO photoanode shows an overlap for current crossing at −0.06 V vs RHE with a current density of around 200 μA cm^–2^ (Figure 2d). This indicates that these two processes (CO_2_ reduction and alcohol oxidation) can be simultaneously driven on CN_*X*_ by sunlight irradiation without any external energy input. This current overlap also provides a meaningful analysis of photocatalyst powder and sheet systems, which effectively represent “short-circuited” photoelectrochemical systems at the nanoscale.

LSV analysis (ν = 10 mV s^–1^) with the final photoelectrochemical assembly in a two-electrode configuration (CN_*X*_–ITO photoanode for MBA oxidation and IO–ITO|FDH cathode for CO_2_ reduction) under continuous and chopped (5s on, 5s off) AM1.5G irradiation shows a catalytic onset voltage at −0.2 V and an average photocurrent density of around 140 μA cm^–2^ at zero applied bias voltage (Figure S18). This indicates that the simulated solar irradiation allows for unassisted and bias-free operation, and is consistent with the three-electrode measurements of the individual (photo)electrodes (Figure 2d). No photocurrent was observed under dark conditions.

Photoelectrolysis experiments under bias-free conditions using CN_*X*_–ITO||IO–ITO|FDH were carried out for 10 h under chopped (50 min light on, 10 min off) and continuous AM1.5G irradiation (Figure 2e). An average photocurrent density of 65 ± 15 μA cm^–2^ was obtained, with a minor decrease in photocurrent density over time that is attributed to slow dissolution of the photocatalyst composite in the electrolyte as well as FDH deactivation.^24^ After 10 h continuous light irradiation, 10.6 ± 0.1 μmol_formate_ cm^–2^ was obtained as a CO_2_ conversion product in the catholyte and 11.1 ± 1.0 μmol_MBAld_ cm^–2^ as an oxidation product in the anolyte. The average faradaic efficiencies of formate and MBAld under chopped and continuous light irradiation were 94 ± 3% for both products, confirming the charge balance of the overall redox process (Figure 2f). No photocurrent was observed when the experiment was performed in the dark.

### Comparison between Different Configurations

**Figure 3:**
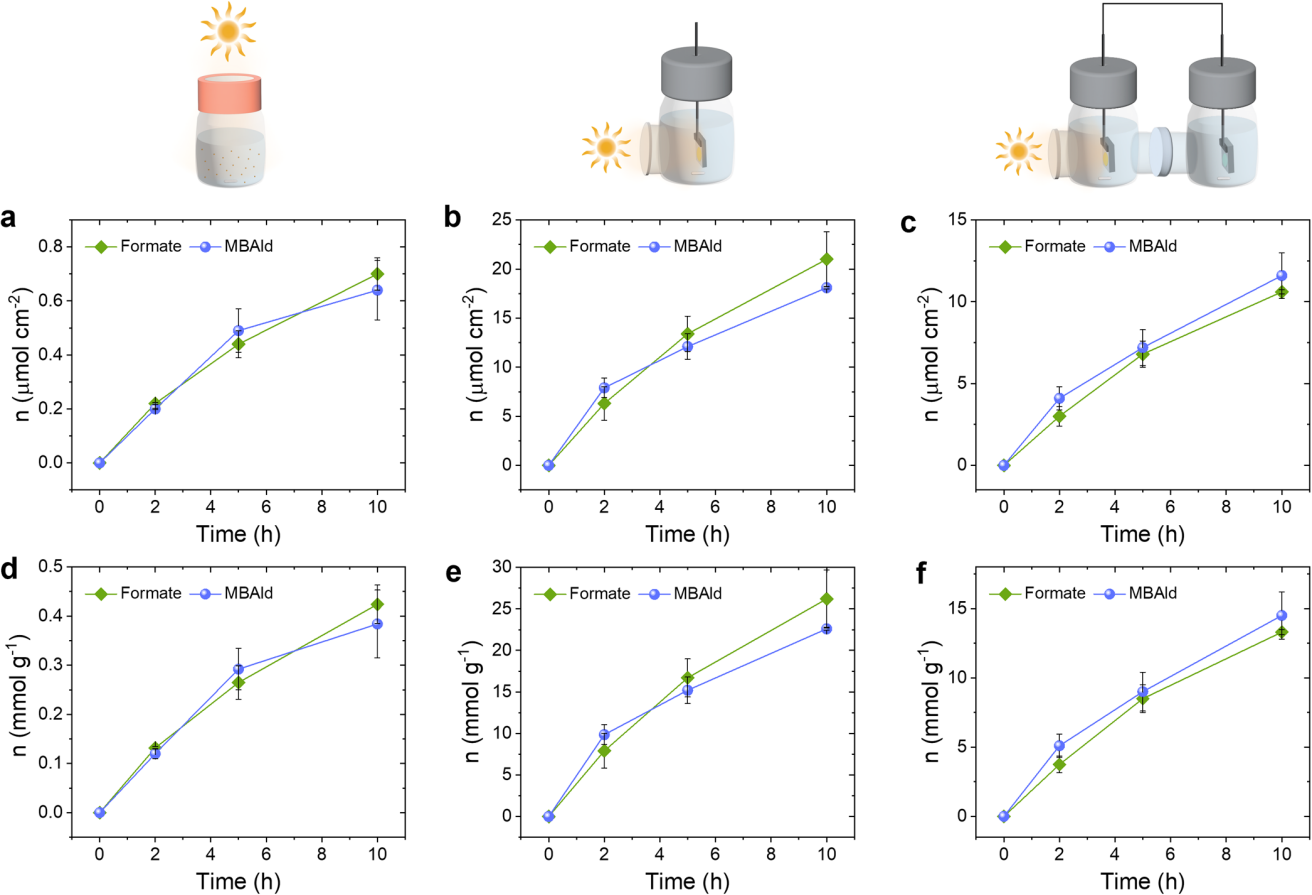
Comparison of different biohybrid systems for solar chemical production. Time-dependent areal activity and activity per g of the photocatalyst composite for (a,d) suspension photocatalyst, (b,e) photocatalyst sheet, and (c,f) 2-electrode photoelectrochemical system. The schematic of each process is presented at the top of the corresponding result for better understanding. The experiments were performed under constant stirring and 1 sun (AM1.5G, 100 mW cm^–2^)-simulated solar irradiation at 30 °C.

After demonstrating the use of CN_*X*_–ITO|FDH in three different configurations, we compared their time-dependent performance (Figure 3). All three configurations show a gradual increase in product production over time, maintaining an approximate 1:1 formate/MBAld ratio. After 10 h, the suspension system produced around 0.7 ± 0.1 μmol_formate_ cm^–2^ (Figure 3a), the photocatalyst sheet 21 ± 3 μmol_formate_ cm^–2^ (Figure 3b), and the bias-free photoelectrochemical cell 10.6 ± 0.1 μmol_formate_ cm^–2^ (Figure 3c). Adjusting for the mass of the photocatalyst composite (CN_*X*_–ITO) results in specific activities of 0.42 ± 0.04 mmol_formate_ (g CN_*X*_–ITO)^–1^ for the suspension system (Figure 3d), 26.2 ± 3.5 mmol_formate_ (g CN_*X*_–ITO)^–1^ for the photocatalyst sheet (Figure 3e), and 13.3 ± 0.17 mmol_formate_ (g CN_*X*_–ITO)^–1^ for the photoelectrochemical cell (Figure 3f). The high specific activity of the photocatalyst sheet and photoelectrochemical cell is due to the small amount of the CN_*X*_–ITO composite (0.8 mg cm^–2^) used to prepare the thin photocatalyst layer, whereas a comparatively high amount (4 mg in 2 mL) of the composite was required for the suspension system.

Despite the overall lower performance of the suspension system, it is a simple platform to perform multiple experiments in parallel. As the suspension system provides an overall comparable trend with the other two immobilized platform setups, it can be easily employed to optimize the biohybrid photocatalyst sheet and photoelectrodes by varying conditions and performing control experiments.

The photocatalyst sheet shows high activity and emerges as an excellent platform for scale-up and performing one-pot reactions under natural sunlight due to its facile fabrication, in agreement with the ongoing development of practical photocatalyst sheets for overall water splitting.^44^ A practical disadvantage is that the single-compartment reaction setup suffers from difficulties in product separation and possibilities of back reactions (note that significant back reactions were not observed in our systems; see the above and Figure S9). The two-compartment photoelectrochemical setup overcomes these issues by separating the oxidative from the reductive reactions by an ion-exchange membrane. However, the separation results in cell resistance (∼10 ± 2 Ω) and mass transport limitations, which reduces the performance of the (photo)electrodes.

The amount of FDH also plays a critical role in the activity of biohybrid assemblies (Figure S10) and different amounts were used in the optimized systems studied here. Importantly, the turnover number (TON) of formate production in all three configurations was in the range of 42,000 to 53,000 after 10 h AM1.5G irradiation (Figure S19). This observation suggests that enzyme activity may have a prominent role in controlling the performance of each system.

The versatility and robustness of the CN_*X*_–ITO|FDH biohybrid system were demonstrated by successfully constructing three different solar-driven configurations. All three systems operated efficiently and resulted in clean catalytic reactions by performing selective CO_2_ reduction and alcohol oxidation processes without any side reactions. The ∼1:1 ratio of oxidation and reduction products demonstrates the charge balance of the overall redox process. Each system has its own advantages and challenges that should be considered when selecting a specific configuration. Considering the performance and practical aspects of each setup, we selected the photocatalyst sheet system for scale-up and testing outdoors under natural sunlight.

### Prototype Scale-Up and Outdoor Testing

**Figure 4:**
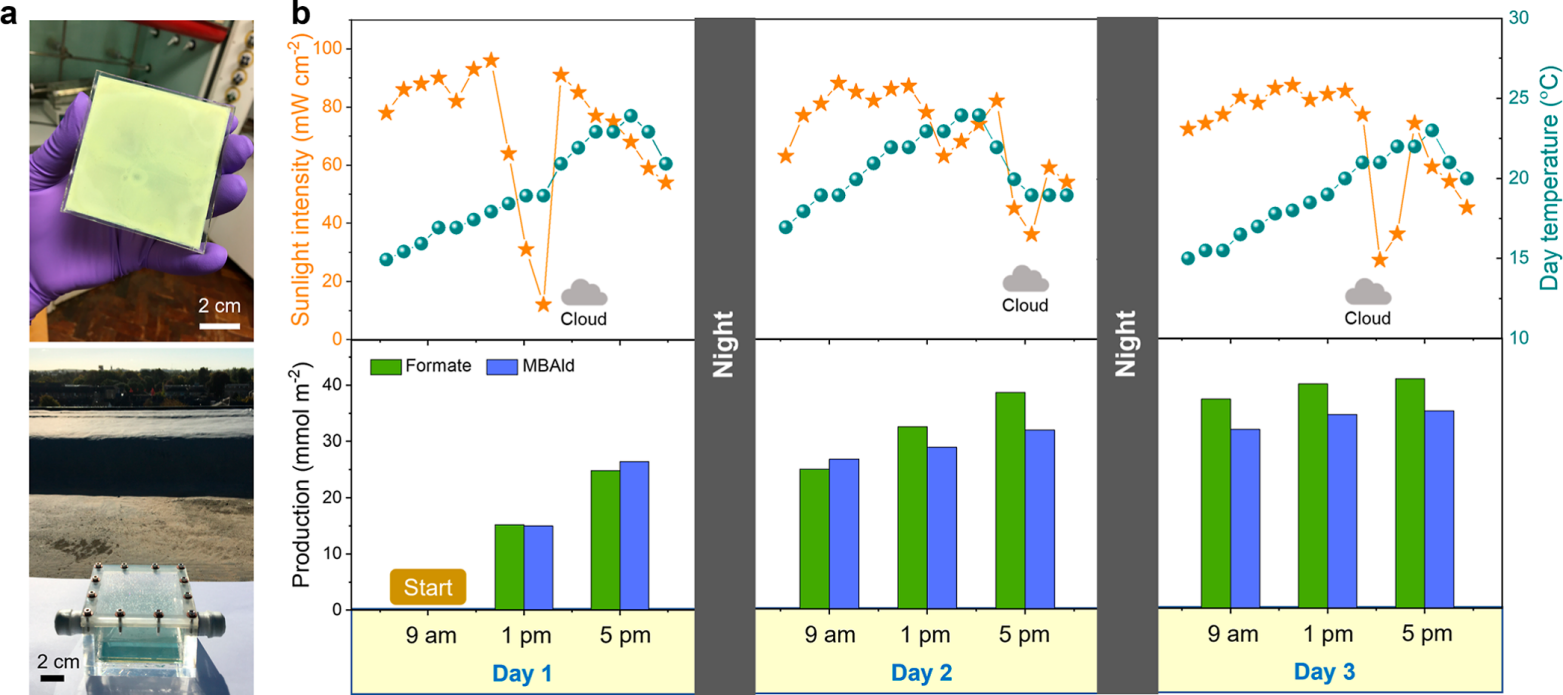
Scaled biohybrid photocatalyst sheet under natural sunlight. (a) Photographs of the 50 cm^2^ CN_*X*_–ITO (1:3) photosheet (fabricated on the FTO substrate, upper panel), and the solar reactor operating under natural sunlight on the rooftop of Yusuf Hamied Department of Chemistry, University of Cambridge (lower panel). (b) Variation of natural-sunlight intensity and day temperature with time (upper panel) and accumulative product formation over time (lower panel).

To demonstrate the scale-up potential and operation under natural sunlight, we developed a 50 cm^2^ CN_*X*_–ITO|FDH photocatalyst sheet using the CN_*X*_–ITO (1:3 w/w) composite (∼40 mg material) on the FTO-coated glass and dropcast FDH (1.6 nmol). To accommodate the 50 cm^2^ photocatalyst sheet, we designed an airtight and transparent Perspex (poly(methyl methacrylate)) photoreactor. FDH immobilization and reactor setup were performed under an inert (N_2_) atmosphere and the photocatalyst sheet was submerged in the CO_2_-saturated solution (see above for detailed composition). The experiment was carried out on the rooftop of the Yusuf Hamied Department of Chemistry, University of Cambridge, for 3 days, from 8^th^ to 10^th^ October 2022 (Figure 4a). Liquid aliquots were taken in regular time intervals for product quantification, and the light intensity and reaction temperature were measured throughout the experiment (Figure 4b, upper panel). The recorded average outside temperature and light intensity were 20 ± 3 °C and 72 ± 22 mW cm^–2^ on day 1, 21 ± 2 °C and 71 ± 16 mW cm^–2^ on day 2, and 19 ± 3 °C and 69 ± 19 mW cm^–2^ on day 3.

The sunlight intensity was on average relatively constant throughout the days except for some short isolated cloudy sessions. The time-dependent product analysis showed a gradual increase in formate and MBAld products in a 1:1 ratio (Figure 4b, lower panel). The 50 cm^2^ biohybrid photocatalyst sheet accumulated 25 mmol_formate_ m^–2^ and 26.5 mmol_MBAld_ m^–2^ at the end of day 1, 38.5 mmol_formate_ m^–2^ and 32 mmol_MBAld_ m^–2^ at the end of day 2, and 41 mmol_formate_ m^–2^ and 35 mmol_MBAld_ m^–2^ at the end of day 3, demonstrating that the biohybrid photosheet was catalytically active over 3 days. While FDH production is currently expensive, this cost can in principle be drastically reduced in the future by using engineered enzymes with specific surface linkers directly from cell lysates without isolation and purification.

Inductively coupled plasma optical emission spectroscopy (ICP-OES) study reveals an increase in the trace of In inside the electrolyte with time, which suggests minor photocatalyst dissolution being one potential reason for the gradual decrease of photocatalytic activity of the biohybrid composite over time (Figure S20). Postcatalysis SEM also shows some cracks and agglomeration in the photocatalyst composite (Figure S21), similar to that observed with the small-size photocatalyst sheet.

## Conclusions

We have systematically developed and studied three different biohybrid systems for solar-driven CO_2_ fixation, demonstrating the versatility of the CN_*X*_–ITO|FDH platform that also enables direct comparison between the three setups. This study thereby shows the possibility to assemble the three most prominent solar fuel configurations, powder suspension, photocatalyst sheet, and photoelectrochemical cell, with the same material components (light absorber, co-catalyst and support/electron relay). This demonstration will encourage the exploration of classical semiconductor powder photocatalysts in photoelectrochemical systems and vice versa and thereby unite approaches and scientists in a more holistic exploration of solar chemical devices. While the three device configurations are currently employed in the development of solar chemistry, they also present promising platforms to develop organic photocatalysis in the future.

All three systems perform well and produce their products cleanly, thus demonstrating high activity and stability in generating the products in the expected stoichiometric ratio. While each setup has its own merits, photocatalyst sheets display high areal and specific activity and allow for simple fabrication, which made it the most suitable choice for assembling a 50 cm^2^ prototype for testing outdoors, the largest integrated semiartificial solar fuel system reported to date. Our demonstration to operate this biohybrid device for selective CO_2_ fixation over continuous 3 days starts to question the common assumption that enzymes cannot be deployed at scale under real-world conditions for solar fuel production and invites more ambitious exploration of their suitability to produce practical devices.

## Experimental Section

### Materials and Chemicals

MBA (≥98% purity), FTO (∼7 Ω sq^–1^)-coated glass, ITO (∼12 Ω sq^–1^)-coated glass, frosted glass slides, ITO nanoparticles (<50 nm diameter, surface area 47 m^2^ g^–1^), and melamine were purchased from Sigma-Aldrich. DL-Dithiothreitol (DTT, Sigma-Aldrich), tris(hydroxymethyl)aminomethane (Tris, Sigma-Aldrich), ^13^C–NaHCO_3_ (Sigma-Aldrich), ^13^CO_2_ (Sigma-Aldrich), H_2_SO_4_ (Fisher Chemicals), sodium hydroxide (Fisher Chemicals), H_2_SO_4_ (Fluka), Na_2_CO_3_, and KCl and NaHCO_3_ (Sigma-Aldrich) were used as received without any further purification. Milli-Q-grade H_2_O was used for all of the experiments. W/Sec-FDH from *Desulfovibrio vulgaris* Hildenborough and NiFeSe H_2_ase from *Desulfovibrio vulgaris* Hildenborough were used as biocatalysts.

### Carbon Nitride Synthesis

Unfunctionalized carbon nitride was synthesized following a previously reported procedure^30^ by heating melamine (5 g) at 550 °C for 3 h (ramp rate 1 °C min^–1^) under air. The obtained yellow powder was ground using a pestle and mortar (yield 50%). CN_*X*_ was synthesized^19^ by mixing the unfunctionalized carbon nitride powder with potassium thiocyanate, in a 1:2 weight ratio, using a pestle and mortar. The mixture was heated for 1 h at 400 °C and then at 500 °C for 30 min (ramp rate 30 °C min^–1^) under an inert atmosphere (Ar). The powder was allowed to cool to room temperature, washed twice with H_2_O, once with H_2_O and ethanol (1:1), and dried overnight at room temperature.

### Synthesis of the Particulate CN_*X*_–ITO Composite

The CN_*X*_–ITO composite was prepared by grinding cyanamide-functionalized CN_*X*_ powder and ITO nanoparticles in a mortar and pestle with different weight ratios (3:1, 1:1, 1:3, and 1:9) and then annealing the mixture in a tube furnace at 250 °C for 1 h under an Ar atmosphere to achieve the photocatalyst composite.

### CN_*X*_–ITO Deposition on Glass Substrates

Carbon nitride was deposited onto an FTO-coated glass substrate following a reported literature procedure,^24^ by codeposition with commercial ITO nanoparticles (diameter <50 nm, surface area 47 m^2^ g^–1^). The CN_*X*_ and ITO powders were mixed in different ratios (3:1, 1:1, 1:3, 1:9, and 25 mg in total), dispersed in ethanol (0.55 mL), and sonicated for 30 min. The FTO side of the glass was covered with a Parafilm mask leaving 0.5 cm^2^ active area for deposition. Two layers of 10 μL of solution were dropcast on the template. The first layer was allowed to dry in air before dropcasting the second one. For the 50 cm^2^ photosheet, the deposition area was masked by a Kapton tape. After the mask was removed, the photosheet/electrode was annealed at 250 °C for 1h 30 min under an inert atmosphere (Ar) and allowed to cool down to room temperature. The CN_*X*_–ITO (1:3) composite was also dropcast on the ITO-coated glass and frosted glass (active area: 0.5 cm^2^) following the same procedure.

### Preparation of IO–ITO Electrodes

The IO–ITO electrodes were prepared following a previously established ITO nanoparticle (<50 nm diameter) and polystyrene bead (750 nm diameter, 2.54% w/v suspension in water) coassembly procedure.^41^ The IO–ITO electrode had a macropore diameter of 750 nm, a film thickness of 20 μm, and a geometric surface area of 0.25 cm^2^.

### FDH Activation and Dropcast

FDH activation and dropcasting were performed under a N_2_ atmosphere in an anaerobic glovebox (MBRAUN). A 40 μM FDH solution in 20 mM Tris buffer at pH 7.6 was added to a freshly prepared 50 mM DTT solution in a 1:1 (v/v) ratio and the mixture was incubated for 30 min. The solution was then diluted to the required FDH amount with 20 mM Tris buffer at pH 7.6 before dropcasting to the CN_*X*_–ITO photocatalyst composites.

### Material Characterization

A TESCAN MIRA3 FEG-SEM instrument equipped with an Oxford Instruments Aztec Energy X-maxN 80 EDX system was used for the SEM and EDX analyses. The TEM measurements, STEM mappings, and high-resolution point EDX analyses were carried out by a Thermo Scientific Talos F200X G2 TEM (FEI, operating voltage 200 kV, 300 mesh Ni grids were used for sample preparation). PXRD measurements were performed by a Panalytical X’Pert Pro (K alpha Cu radiation) diffractometer using a 1° min^–1^ scan rate. ICP-OES measurements were performed by using a Thermo Scientific iCAP 7400 ICP-OES DUO spectrometer.

### Photocatalysis and Photoelectrocatalysis Experiments

After immobilizing the activated FDH enzyme (40 pmol), the 4 mg powder photocatalyst composites (CN_*X*_–ITO ratios 1:0, 3:1, 1:1, 1:3, 1:9, and 0:1) were suspended in 2 mL CO_2_-saturated 100 mM NaHCO_3_ buffer containing 50 mM KCl and 7.5 mM MBA (pH 6.7) in top-down reactors. Simulated sunlight (1 sun, 100 mW cm^–2^, AM1.5G) was irradiated from the top and the experiments were carried out for 10 h at 30 °C. The reactor setups were performed in an MBraun glovebox under a N_2_ atmosphere and the photochemical experiments were performed outside the glovebox with airtight sealed reactors.

Single-compartment reactors were used for the experiments with the photocatalyst sheets. 200 pmol FDH was dropcast on a 0.5 cm^2^ surface area of the CN_*X*_–ITO photosheets (with different CN_*X*_–ITO ratios 1:0, 3:1, 1:1, 1:3, 1:9, and 0:1). For FDH concentration optimization, 50, 100, 200, and 300 pmol FDH were dropcast on the CN_*X*_–ITO (1:3) photosheet. A CO_2_-saturated 100 mM NaHCO_3_ buffer containing 50 mM KCl and 7.5 mM MBA (pH 6.7) was used as the reaction medium (total volume: 10 mL) and the photosheets were irradiated from the front. The experiments were carried out under simulated solar irradiation (1 sun, 100 mW cm^–2^, AM1.5G) at 30 °C. For the prototype scalable photosheet experiment, an 8 cm × 8 cm × 5 cm airtight Perspex (poly(methyl methacrylate)) photoreactor was designed. The 50 cm^2^ photocatalyst sheet experiment was performed on the rooftop of the Yusuf Hamied Department of Chemistry for 3 days (8^th^, 9^th^, and 10^th^ October, 2022) under natural sunlight. The sunlight intensity and the reactor temperature were monitored from time to time with a certified Newport 1916 R optical power meter and a laboratory thermometer, respectively. The reactors were assembled inside a glovebox under a N_2_ atmosphere.

The photoelectrochemical experiments were performed in a 2-compartment reactor where the photoanode and cathode compartments were separated by a proton exchange membrane (Nafion^117^, Fuel Cell Stores). A CN_*X*_–ITO (1:3 ratio) photoelectrode was used as the photoanode for MBA oxidation. An inverse-opal ITO electrode (0.25 cm^2^ surface area, deposited on an FTO-coated glass substrate) was used as a substrate where 100 pmol FDH was dropcast to prepare the cathode. CO_2_-saturated 100 mM NaHCO_3_ with 50 mM KCl (5 mL, pH 6.7) was used as a catholyte and CO_2_-saturated 100 mM NaHCO_3_ with 50 mM KCl containing 7.5 mM MBA (15 mL, pH 6.7) was used as an anolyte. The reactor setups were prepared in a glovebox under a N_2_ atmosphere.

The cyclic voltammograms were measured with a scan rate of 10 mV s^–1^ under chopped, continuous, and no solar (1 sun, AM1.5G, 100 mW cm^–2^) irradiation. The potentials during the 3-electrode experiments were measured against a Ag/AgCl (saturated NaCl) reference electrode (a Pt mesh was used as a counter electrode) and were converted to the RHE scale using the equation
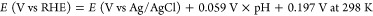


The chronoamperometry experiments were also performed under chopped (50 min light on, 10 min off), continuous, and no simulated solar irradiation without external bias voltage. All photoelectrochemical experiments were performed at 30 °C.

### H_2_ase-Based Biohybrid Assembly and Photocatalysis

H_2_ase (100 pmol) was dropcast on the CN_*X*_–ITO (5 mg, 1:3 wt ratio) photocatalyst under a N_2_ atmosphere inside a glovebox and added to a CO_2_-saturated NaHCO_3_ aqueous solution (100 mM) containing KCl (50 mM) and MBA (2 mM) or formate (2 mM) at pH 6.7 in a top-down photoreactor. No MBA or formate was added to the reaction medium when water was used as the electron donor. Simulated sunlight (1 sun, 100 mW cm^–2^, AM1.5G) was irradiated from the top and the experiments were carried out for 8 h at 30 °C.

### Product Quantification

High-performance liquid chromatography (HPLC) and ion-exchange chromatography (IC) were used to quantify the liquid products. HPLC analyses were performed using a Waters Breeze system equipped with refractive index (RID-2414) and diode array UV–vis (λ = 210 and 254 nm) detectors. Identification and quantification of the MBA oxidation product were carried out using a C18 column at a column temperature of 40 °C. The samples were analyzed in the isocratic flow mode with a flow rate of 0.5 mL min^–1^ using a 1:1 (v/v) mixture of H_2_O and MeCN. Formate was quantified by ion chromatography. The analysis was performed with an 882 Metrohm Compact Ion Chromatograph Plus using 3.2 mM Na_2_CO_3_ and 1 mM NaHCO_3_ as an eluent. Calibration was conducted with external standards for both processes. The headspace of the reactors was injected into a Shimadzu GC-2010 Plus gas chromatograph equipped with a PLOT-MS 5 A Molsieve column for H_2_ detection. Negligible amounts or no H_2_ was detected after the experiments indicating that the FDH selectively performs the CO_2_-to-formate conversion.

## Data Availability

The raw data underpinning the findings of this study can be accessed through the University of Cambridge data repository: https://doi.org/10.17863/CAM.116141.

## Supporting Information Available

Material characterization via TEM, UV–vis, SEM, STEM–EDX, and XRD analyses; photographs of different systems; performance optimization varying different parameters; photocatalysis with the H_2_ase biohybrid; recyclability tests; electrochemical and photoelectrochemical characterizations; turnover number calculation; time-dependent ICP-OES analysis; and postcatalysis analyses (PDF)
