# Supplementary material for: Solar-Driven Paired CO2 Reduction–Alcohol Oxidation Using Semiartificial Suspension, Photocatalyst Sheet, and Photoelectrochemical Devices
Source: J Am Chem Soc. 2025 Feb 28;147(10):8168–77. doi: 10.1021/jacs.4c10519 (PMC11912307; doi:10.1021/jacs.4c10519)
Supplement: Supplementary file 1 — ja4c10519_si_001.pdf [file ja4c10519_si_001.pdf]

Supporting Information

Solar-driven paired CO_2 reduction-alcohol oxidation using semiartificial suspension, photocatalyst sheet, and photoelectrochemical devices

Motiar Rahaman,[a] Carolina Pulignani,[a] Melanie Miller,[a] Subhajit Bhattacharjee,[a] Ariffin Bin Mohamad Annuar,[a] Rita R. Manuel,[b] Inês A. C. Pereira,[b] and Erwin Reisner[a,*]

[a]*Yusuf Hamied Department of Chemistry, University of Cambridge, Lensfield Road, Cambridge CB2 1EW, United Kingdom*
[b]*Instituto de Tecnologia Química e Biológica António Xavier (ITQB NOVA), Universidade NOVA de Lisboa, 2780-157 Oeiras, Portugal*

***Corresponding author**

Professor Erwin Reisner

Postal address: *Yusuf Hamied Department of Chemistry, University of Cambridge, Lensfield Road, Cambridge CB2 1EW, United Kingdom*

E-mail: reisner@ch.cam.ac.uk

Tel: +44-1223336323

Website: http://www-reisner.ch.cam.ac.uk/

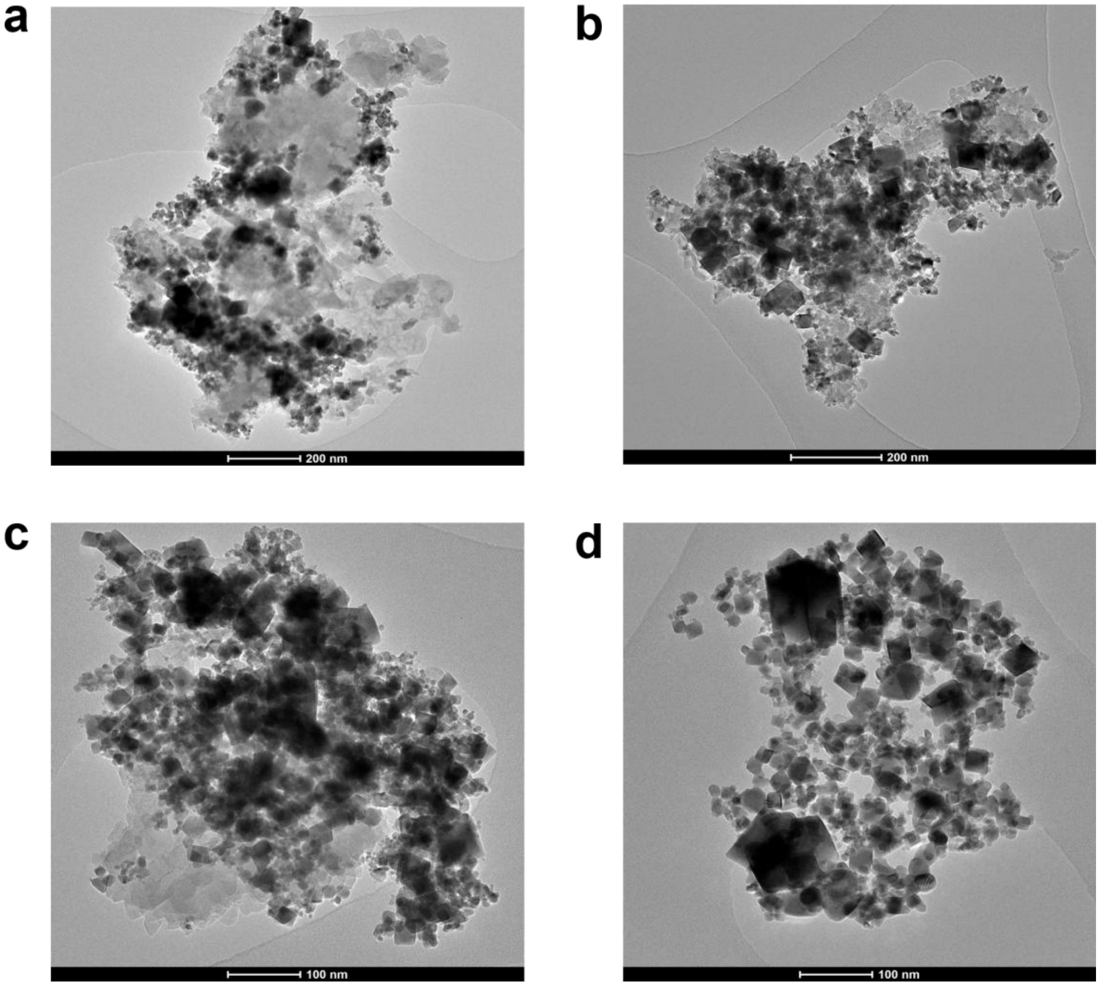

Figure S1. TEM analysis with CN_X-ITO composites: **a,** CN_X-ITO (3:1), **b,** CN_X-ITO (1:1), **c,** CN_X-ITO (1:3), and **d,** CN_X-ITO (1:9) composites.

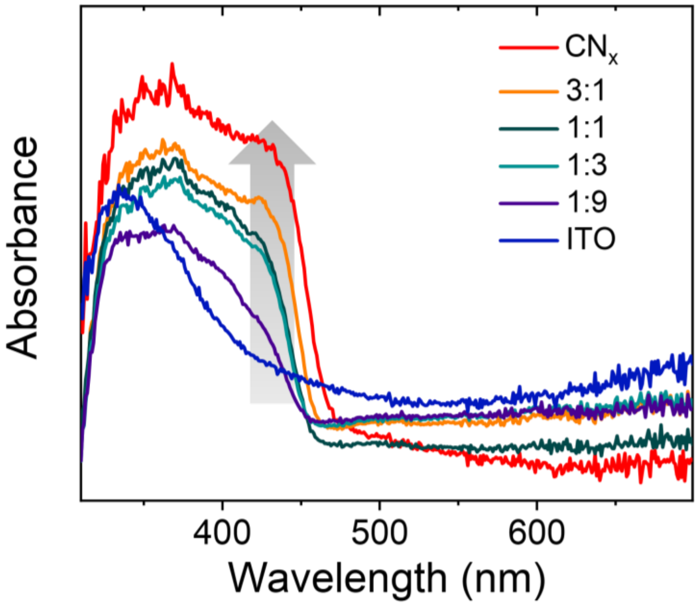

Figure S2. UV-vis spectroscopy of CN_X-ITO composites: The increase of carbon nitride absorption peak is highlighted with an arrow.

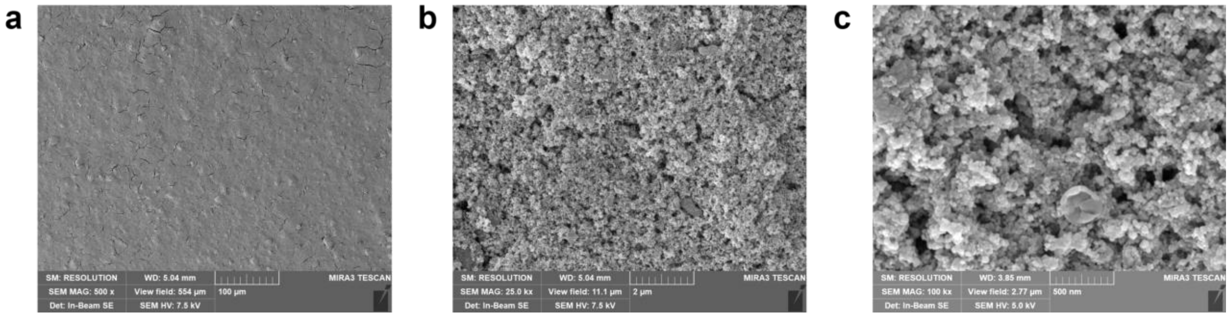

Figure S3. SEM analysis with CN_X-ITO (1:3) composite: Low to high magnification SEM images of a CN_X-ITO (1:3) photosheet.

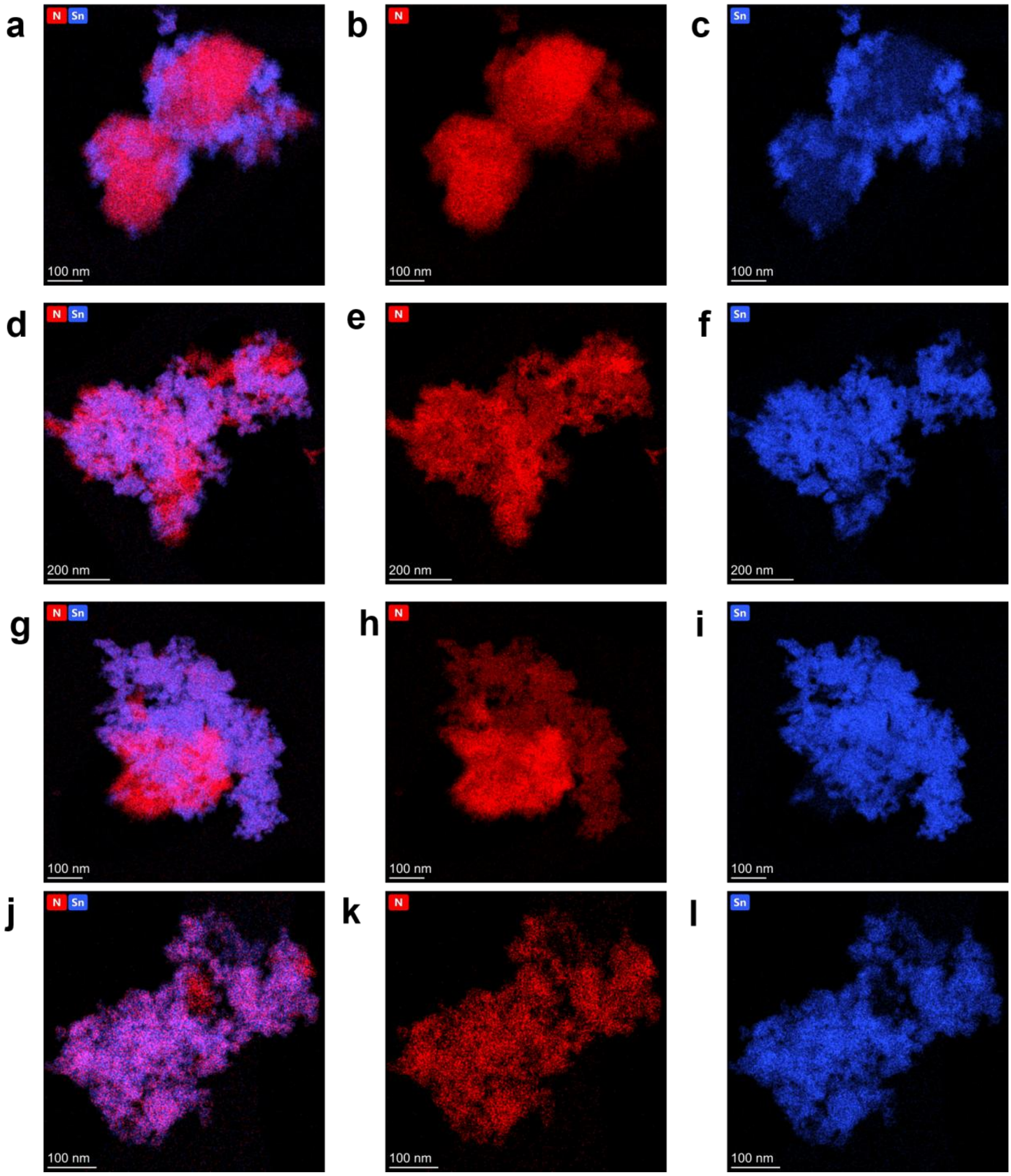

Figure S4. STEM-EDX mapping with different compositions: STEM-EDX mapping with **a-c,** CN_X-ITO (3:1)**, d-f,** CN_X-ITO (1:1), **g-i,** CN_X-ITO (1:3), and **j-l,** CN_X-ITO (1:9) showing the distribution of CN_X and ITO components. Element N (red) comes from CN_X and Sn (blue) comes from ITO. The first column shows joint N and Sn mapping, the second column shows N mapping, and the third column shows the Sn mapping.

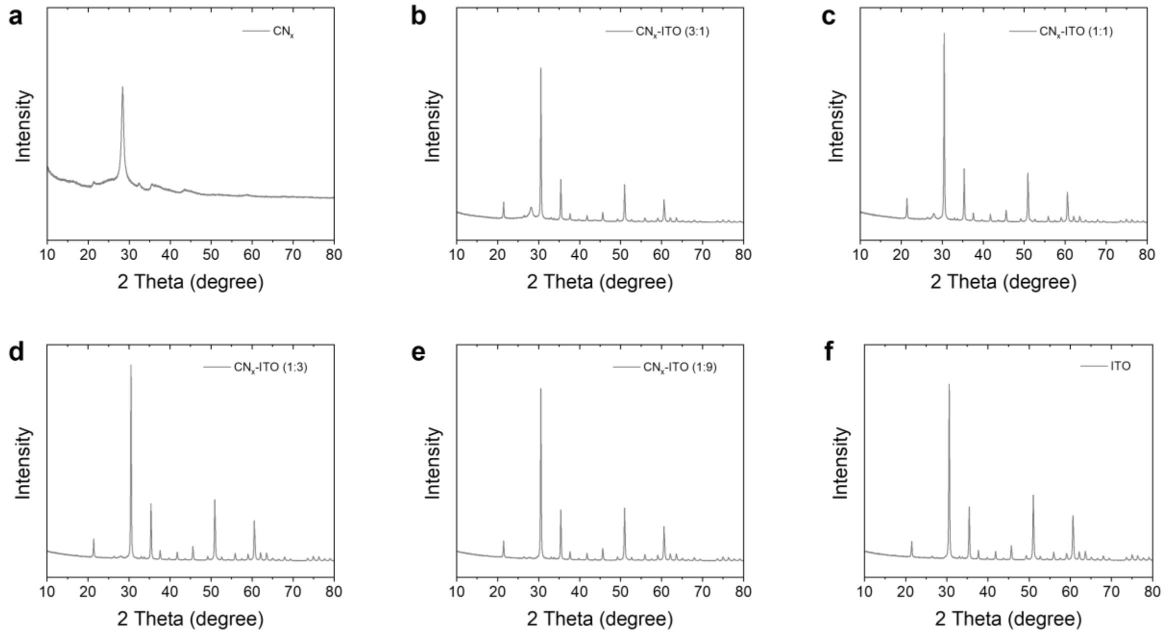

Figure S5. Powder XRD analysis with CN_X, ITO and CN_X-ITO photocatalyst composites. **a,** CN_X, **b,** CN_X-ITO (3:1)**, c,** CN_X-ITO (1:1), **d,** CN_X-ITO (1:3), **e,** CN_X-ITO (1:9), and **f,** ITO. The peak at 2θ 28.1° corresponds to the characteristic XRD pattern of carbon nitride and the peak at 2θ 31° corresponds to the (222) plane of the ITO nanoparticles.

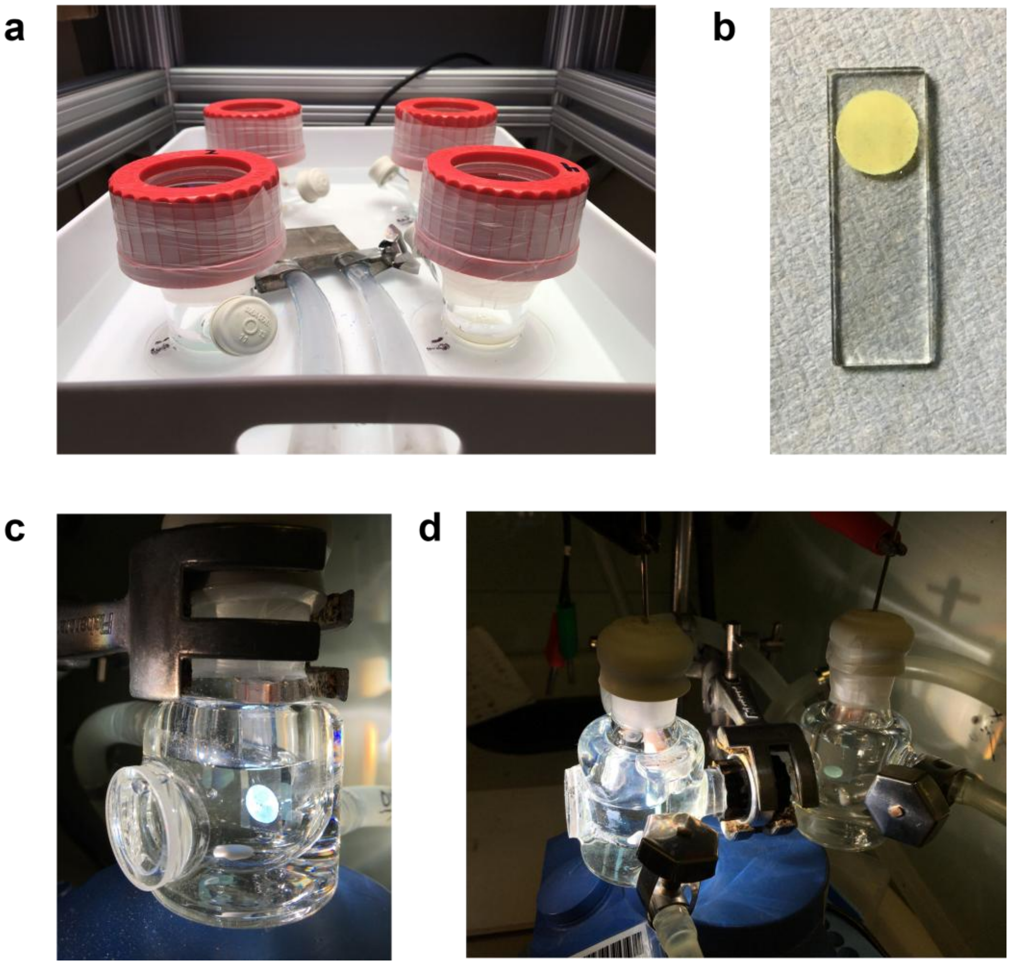

Figure S6. Photographs of different solar-driven systems. **a,** Photograph of a set of 4 experiments with suspension photocatalysts with top-down solar irradiation**. b,** Photosheet with 0.5 cm^2 active surface area. **c,** Photosheet under operation in a single compartment cell. **d,** 2-compartment 2-electrode photoelectrochemical system under operation.

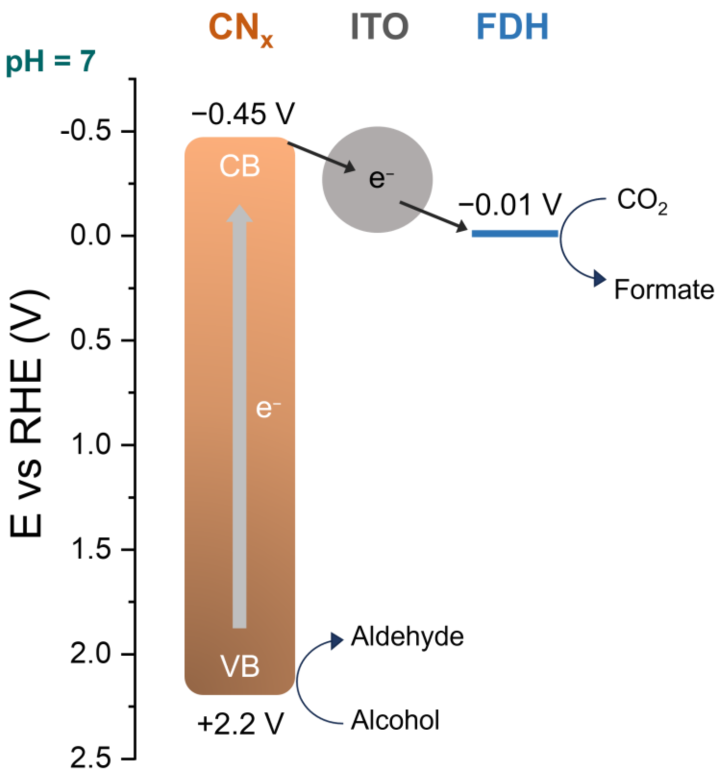

Figure S7. Band structure and charge transfer process of the biohybrid assembly under solar irradiation. A schematic diagram shows the valance band (VB) and conduction band (CB) position of the carbon nitride (CN_X) photocatalyst. The ITO nanoparticles work as support materials for FDH immobilization and conductive connectors to transport electrons from the CB of CN_X to the FDH biocatalyst. The band position of the semiconductor photocatalyst and the thermodynamic standard potential required for the CO_2 conversion process over FDH indicate a facile electron transfer process to accomplish the reaction.

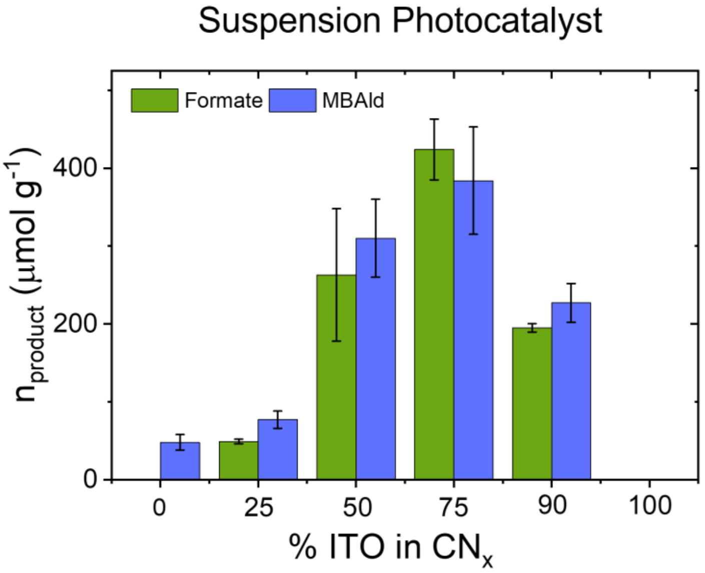

Figure S8. Activity per g of photocatalyst composite. Composition-dependent activity of suspension photocatalyst systems.

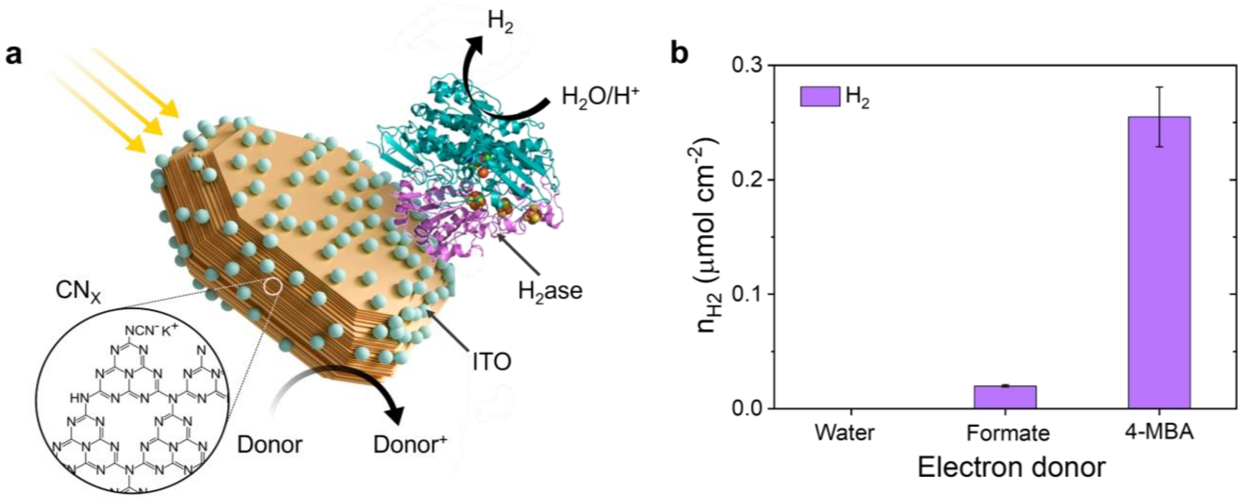

Figure S9. Photocatalytic H_2 evolution using different electron donors. **a,** Schematic diagram of CN_x-ITO|H_2ase biohybrid assembly for solar H_2 production. **b,** Performance of the biohybrid photocatalyst system towards H_2 evolution with different electron donors. The experiments were carried out for 8 h under 1 sun irradiation at 30 °C. No H_2 or negligible H_2 evolution was observed when water or formate was used as electron donor indicating the unfeasibility of the water or formate oxidation process under the experimental conditions. The biohybrid assembly produced substantial H_2 in the presence of MBA as an electron donor.

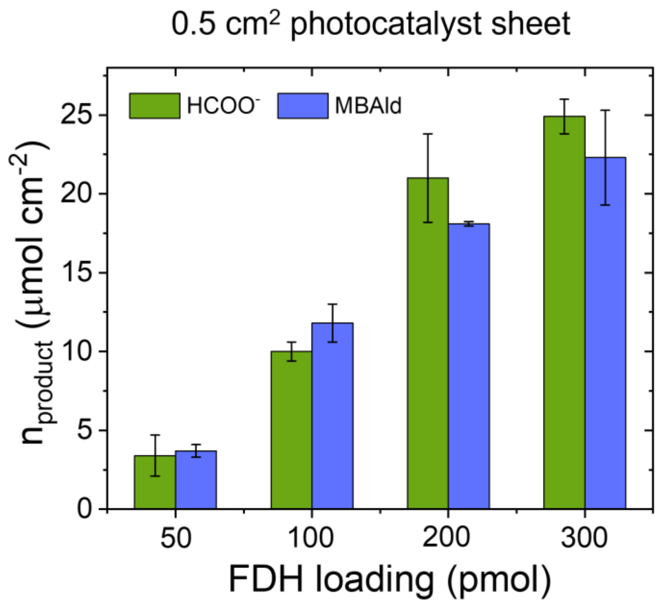

Figure S10. Optimisation of FDH loading. FDH loading on the CN_X-ITO (1:3) photocatalyst sheet where 200 pmol FDH appears to be the best biocatalyst loading.

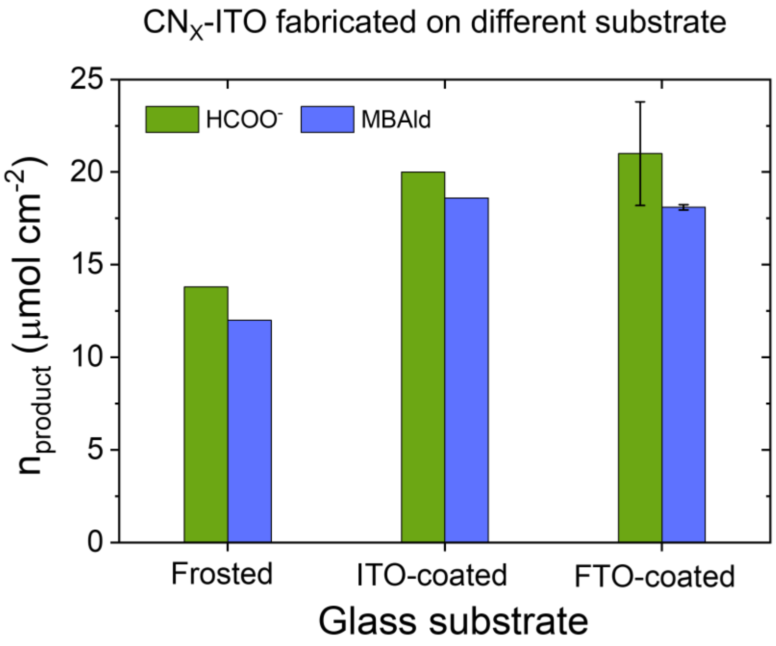

Figure S11. Photocatalyst sheet fabricated on different substrates. Activity comparison of a CN_X-ITO(1:3)|FDH(200 pmol) biohybrid composite fabricated on frosted, ITO-coated, and FTO-coated glass substrates where ITO- and FTO-coated glass demonstrated similar activity but the frosted glass showed some degraded activity due to mechanical instability. The experiments were performed for 10 h under 1 sun irradiation at 30 °C.

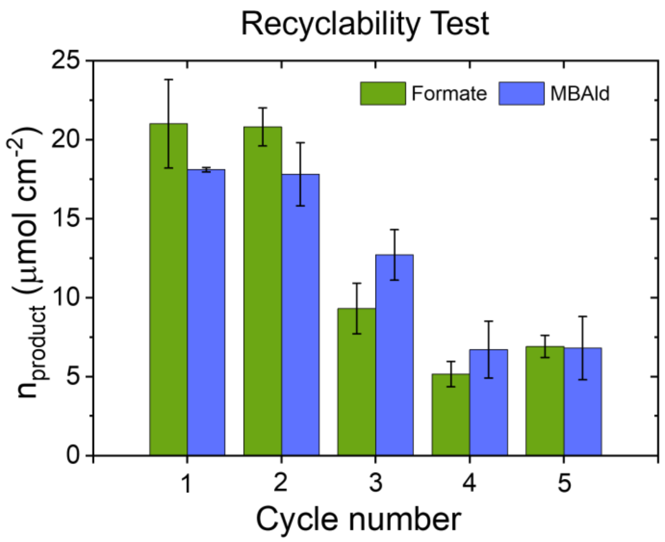

Figure S12. Robustness of a photocatalyst sheet. Experiments showing multiple runs where FDH was reloaded on the same photocatalyst sheet after each 10 h experiment. After the 4th cycle, the photosheet was further cleaned by an ozone treatment to remove any residual dead FDH from the surface.

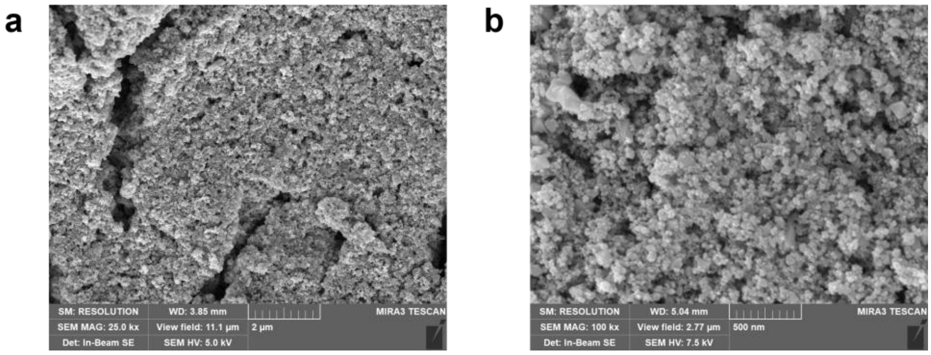

Figure S13. Post-catalysis SEM analysis. **a, b,** Low and high-resolution SEM analyses of CN_X-ITO photosheet after 10 h experiment showing bigger cracks due to dissolution during the experiment.

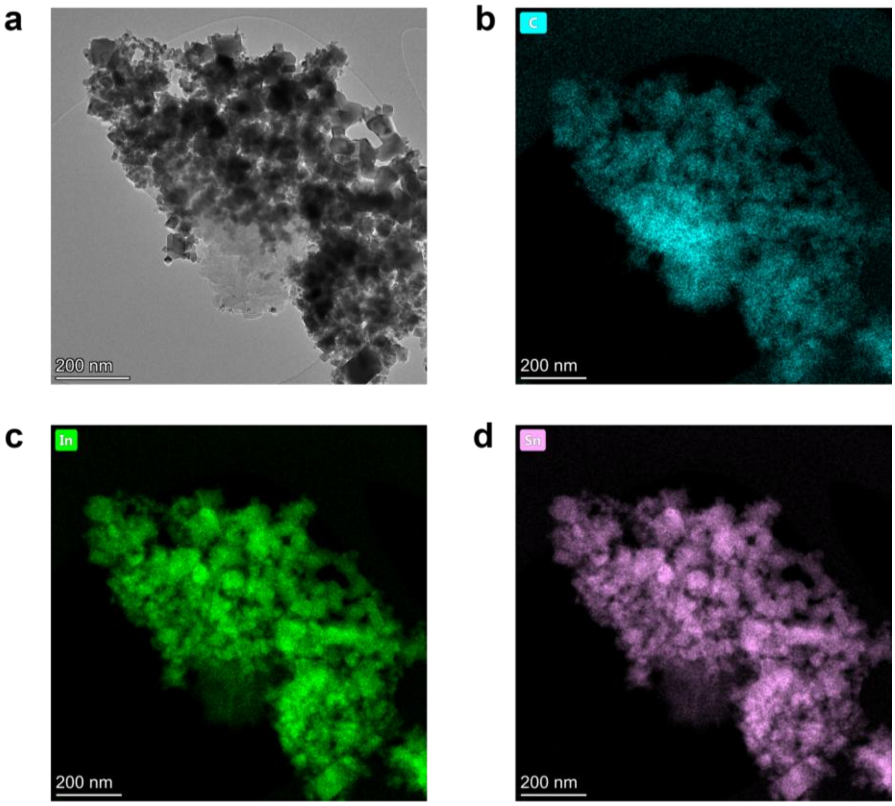

Figure S14. Post-catalysis (after 10 h experiment) TEM and STEM analyses show agglomeration in the nanostructures. **a,** Post catalysis TEM analysis, **b-d,** STEM mapping of different components **b,** C from CN_X, **c,** In from ITO, and **d,** Sn from ITO.

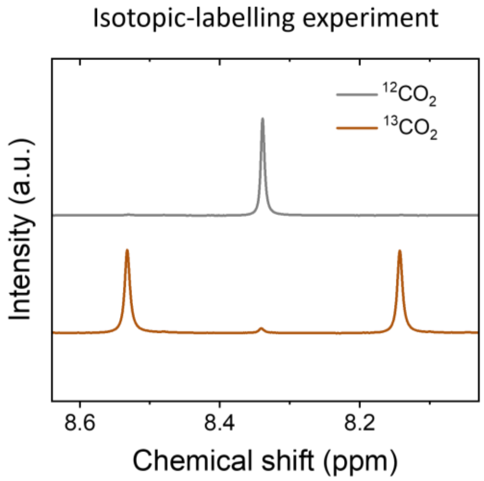

Figure S15. Isotopic labelling experiments with $NaH^{13/12}CO_3$ and $^{13/12}CO_2$, showing the origin of formate being actually from CO_2. CN_X-ITO(1:3)|FDH(200 pmol) biohybrid photosheets were used for the isotopic labelling experiments. ^1H-NMR spectroscopy analyses were carried out for the $H^{12}COO^-$ and $H^{13}COO^-$ detection where a peak splitting was observed in case of $H^{13}COO^-$. The experiments were carried out at 30 °C.

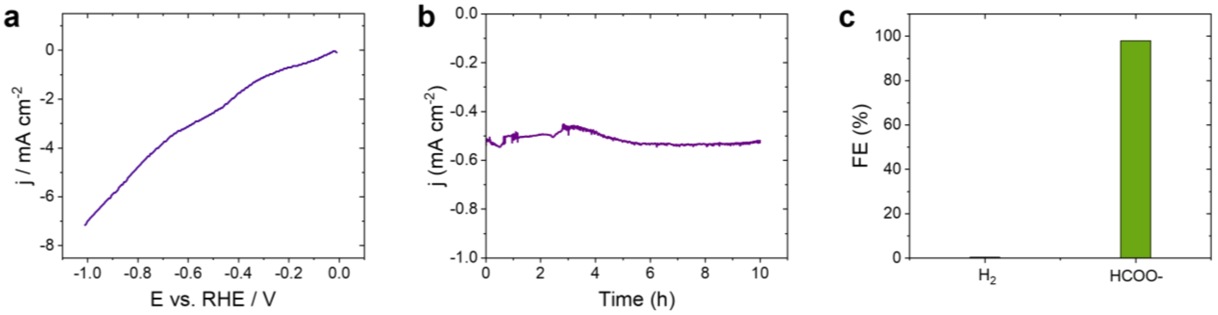

Figure S16. Electrochemistry with FDH. **a,** Linear sweep voltammetry (LSV, 3-electrode system; IO-ITO|FDH as a working electrode, Ag/AgCl as a reference, and Pt mesh as a counter electrode in a 2-compartment reactor) shows an early onset of FDH for the CO_2 electrolysis process. **b,** Current transient of CO_2 electrolysis conducted at −0.09 V vs. RHE. **c,** Faradaic efficiency of formate as the only product shows the catalyst selectivity. CO_2-saturated 0.1 M $NaHCO_3$ with 0.05 M KCl (pH 6.7) was used as an electrolyte.

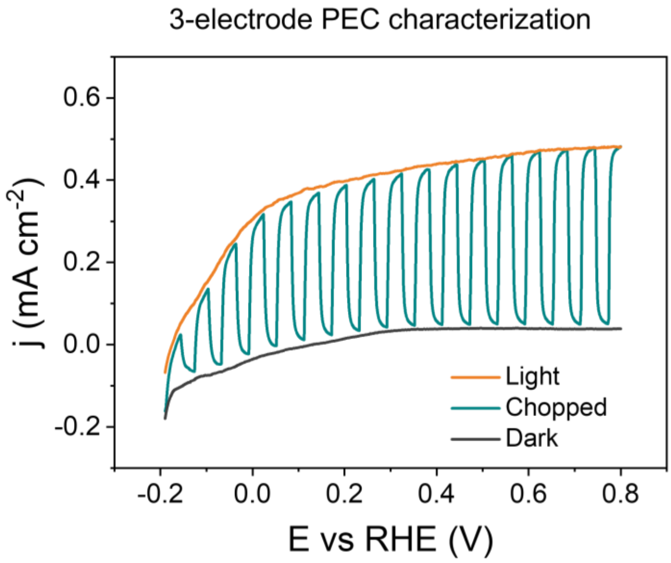

Figure S17. Photoelectrochemical characterization of MBA oxidation process. Linear sweep voltammetry (LSV) scans using a 3-electrode system with a CNx-ITO(1:3) photoanode working electrode, a Pt mesh counter electrode, and an Ag/AgCl reference electrode under chopped, continuous and no simulated solar-light illumination (AM 1.5 G; scan rate: 10 mV s^{-1}). A two-compartment photoelectrochemical reactor separated by a Nafion membrane was used. An aqueous solution of 0.1 M $NaHCO_3$ and 0.05 M KCl (CO_2 saturated, pH 6.7) containing 0.02 M MBA was used as an electrolyte. An onset of the MBA oxidation process at −0.2 V vs RHE with a steady state photocurrent of ~0.45 mA cm^{-2} indicates excellent catalytic efficiency of CNx-ITO photoanode towards the alcohol oxidation process. PEC: photoelectrochemical.

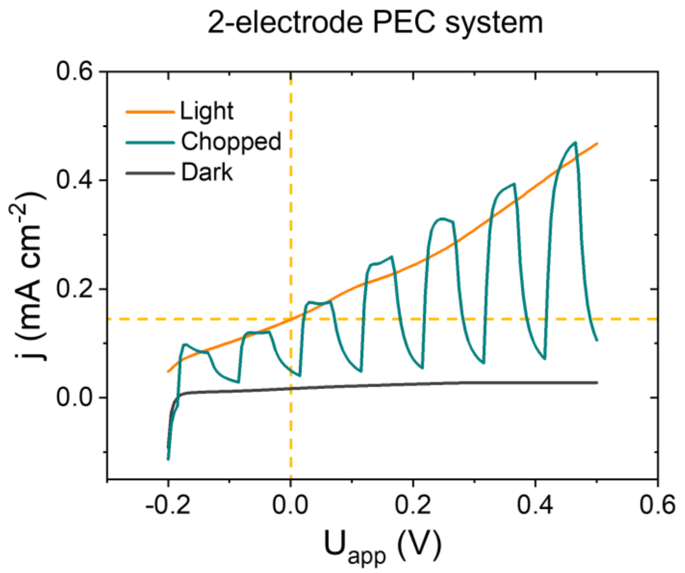

Figure S18. LSV with 2-electrode photoelectrochemical system. LSV under chopped, continuous, and dark conditions with a scan rate of 10 mV s^{-1}. 1 sun (AM1.5G, 100 mW cm^{-2}) simulated solar irradiation was used and the experiment was carried out at 30 °C. A photocurrent of ~0.14 mA cm^{-2} was observed at 0 applied bias indicating the system's ability for unassisted bias-free operation.

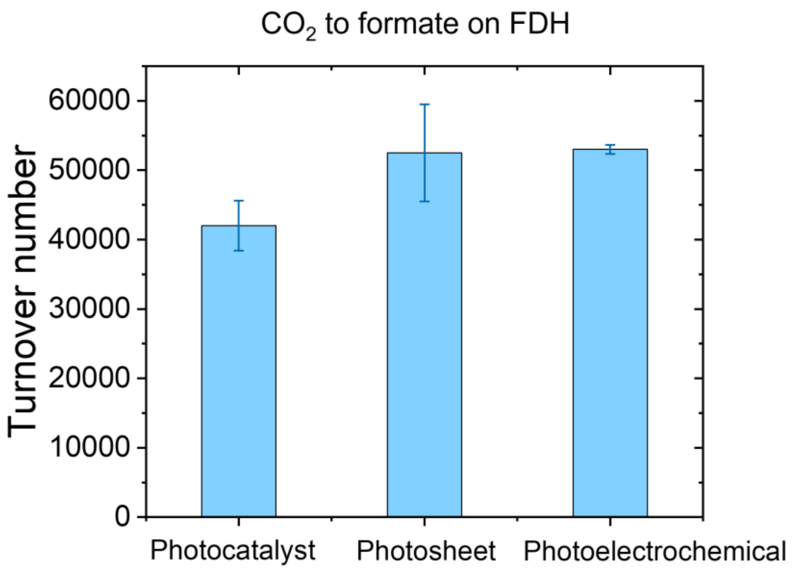

Figure S19. Turnover number of CO_2 to formate formation catalysed by FDH for three different configurations.

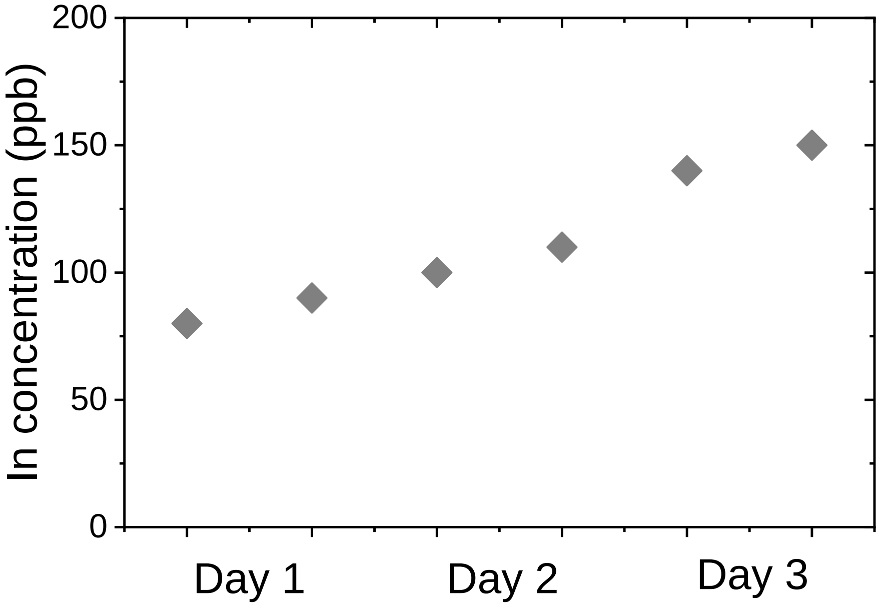

Figure S20. Time-dependent ICP-OES analysis with the prototype photosheet. In content in the solution with time during the experiment with 50 cm^2 photocatalyst sheet under natural sunlight.

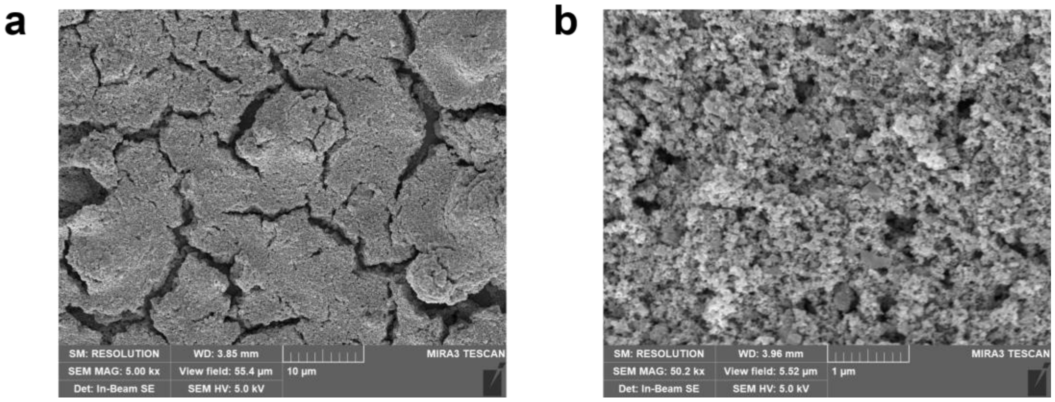

Figure S21. Post-catalysis SEM analysis with the prototype photosheet. SEM analysis with low (a) and high (b) resolution with the prototype 50 cm^2 photocatalyst sheet after 3 days of operation under natural sunlight.

Table S1. Control experiments with the biohybrid photosheet show the feasibility of the process.

Sl No.	CN_x	ITO	FDH	CO_2	4-MBA	Light	Result
**1**	No	Yes	Yes	Yes	Yes	Yes	Negative
**2**	Yes	No	Yes	Yes	Yes	Yes	Negative*
**3**	Yes	Yes	No	Yes	Yes	Yes	Negative*
**4**	Yes	Yes	Yes	No	Yes	Yes	Negative*
**5**	Yes	Yes	Yes	Yes	No	Yes	Negative
**6**	Yes	Yes	Yes	Yes	Yes	No	Negative
**7**	Yes	Yes	Yes	Yes	Yes	Yes	Positive

Experiment conditions: Control experiments were performed both with suspension photocatalyst and photocatalyst sheet. The optimised composition CN_X-ITO (1:3) was used for the control experiments.

Photocatalyst experiments: 4 mg of photocatalyst composites, 2 mL CO_2-saturated reaction medium containing 7.5 mM MBA, 40 pmol FDH, 1 sun (AM1.5G, 100 mW cm^{-2}) irradiation.

Photosheet experiments: 0.5 cm^2 photosheet area, 10 mL CO_2-saturated reaction medium containing 7.5 mM MBA, 200 pmol FDH, 1 sun (AM1.5G, 100 mW cm^{-2}) irradiation.

*Trace amount of 4-MBAld production was observed due to the charge accumulation capacity of CN_X under sunlight irradiation.

END OF SUPPORTING INFORMATION
